# Molecular characterization and resistance mechanisms of ertapenem-non-susceptible carbapenem-resistant *Klebsiella pneumoniae* co-harboring ESBLs or AmpC enzymes with porin loss or efflux pump overexpression

**DOI:** 10.1128/jb.00148-25

**Published:** 2025-06-13

**Authors:** Yingying Su, Guangmei Zou, Xin Huang, Jinli Bi, Liqin Meng, Wei Zhao, Taijie Li

**Affiliations:** 1 Department of Clinical Laboratory, Wuming Hospital of Guangxi Medical University 669939, Nanning, China; 2 Department of Clinical Laboratory, The First People’s Hospital of Yulin, Yulin, China; 3 Department of Oncology, Wuming Hospital of Guangxi Medical University, Nanning, China; University of Virginia School of Medicine, Charlottesville, Virginia, USA

**Keywords:** carbapenem-resistant *Klebsiella pneumonia*, mechanisms of drug resistance, carbapenemase, outer membrane porin, efflux pump

## Abstract

Carbapenem-resistant *Klebsiella pneumoniae* (CRKP) poses a significant challenge in clinical settings due to limited treatment options and high mortality rates. While carbapenemase-producing CRKP has been extensively studied, the mechanisms underlying resistance in non-carbapenemase-producing CRKP remain less understood. In this study, we investigated 30 ertapenem-resistant non-carbapenemase-producing CRKP clinical isolates. Antimicrobial susceptibility testing revealed multidrug resistance in all strains. Molecular analyses showed that all isolates carried extended-spectrum β-lactamase (ESBL) and/or AmpC β-lactamase genes. Sequencing of outer membrane porin (OMP) genes revealed mutations or deletions in *ompK35* and/or *ompK36* in the majority of isolates. SDS-PAGE analysis confirmed reduced or absent expression of the corresponding OMP proteins in these strains. Additionally, quantitative PCR showed that several isolates exhibited overexpression of efflux pump genes. These findings indicate that the combined effects of ESBL or AmpC production, porin loss at both the genetic and protein levels, and efflux pump overactivity contribute to ertapenem resistance in non-carbapenemase-producing CRKP. Our results underscore the complexity of resistance mechanisms and highlight the importance of integrated molecular and phenotypic assessments to inform appropriate antimicrobial therapy.

**IMPORTANCE:**

This study highlights the complex, multifactorial nature of carbapenem resistance in carbapenem-resistant *Klebsiella pneumoniae* (CRKP), involving enzyme-mediated resistance, reduced membrane porin expression, and overactive efflux pumps. These findings provide valuable insights into CRKP resistance mechanisms and can aid in controlling CRKP in China.

## INTRODUCTION

*Klebsiella pneumoniae* (KP) are short bacilli that can exist alone or in pairs, are structured with a thick cellulose membrane, can ferment lactose, are Gram-negative, and are also partially anaerobic (1). KP usually inhabit the gastrointestinal, skin, respiratory, and urinary tracts of humans. When these bacteria release their virulence factors, such as capsules, pili, and siderophores, or iron carriers, they may cause various types of diseases (2), including life-threatening infections, such as cystitis, pneumonia, parenteral infections, endocarditis, and sepsis. They are also the most common causative agents of hospital-acquired infections (3).

In recent years, the increasing incidence of carbapenem-resistant *Klebsiella pneumoniae* (CRKP) has emerged as a serious global public health concern (4). Carbapenems are considered the last-resort antibiotics for treating severe infections caused by multidrug-resistant Gram-negative bacteria, including extended-spectrum β-lactamase (ESBL)-producing strains. However, widespread use of carbapenems has led to the emergence of resistant strains, which often display co-resistance to other antimicrobial classes such as aminoglycosides and fluoroquinolones, leaving limited treatment options (5). Carbapenem resistance in *Klebsiella pneumoniae* is primarily mediated by the production of carbapenemases. However, other mechanisms, including overexpression of ESBLs or AmpC β-lactamases, loss or mutation of outer membrane porins, overactivity of efflux pumps, biofilm formation, and persistence phenotypes, can also contribute to resistance (6). Notably, an increasing number of studies have reported the presence of carbapenem-resistant *Klebsiella pneumoniae* strains that do not produce any known carbapenemases. These strains are collectively referred to as non-carbapenemase-producing carbapenem-resistant *Klebsiella pneumoniae*.

Compared with carbapenemase-producing strains, non-carbapenemase-producing carbapenem-resistant *Klebsiella pneumoniae* remains under-characterized. These strains typically acquire resistance through a combination of ESBL and/or AmpC enzyme production together with outer membrane porin loss or dysfunction and efflux pump overexpression (7). Among clinically relevant carbapenems, ertapenem is considered the most sensitive to permeability-related resistance mechanisms, particularly porin loss. The growing clinical detection of such strains, especially in high-risk departments, such as intensive care units and organ transplantation wards, poses additional challenges for diagnosis, treatment, and infection control.

In this study, we aimed to investigate the molecular characteristics and resistance mechanisms of non-carbapenemase-producing carbapenem-resistant *Klebsiella pneumoniae* strains isolated from tertiary hospitals in Nanning, China. By analyzing antimicrobial susceptibility profiles, resistance gene carriage, porin gene alterations, efflux pump expression, and outer membrane protein levels, this study seeks to provide comprehensive insights into the resistance profiles of these clinically important pathogens and support more effective infection control strategies.

## MATERIALS AND METHODS

### Source of strains

A total of 138 non-duplicate strains of CRKP were collected from four tertiary general hospitals in the Nanning region between 2020 and 2023. After carbapenemase testing (see below), 30 strains were included in this study. Informed consent was obtained from the patients. The inclusion criteria required identification as KP, and the sensitivity of the bacteria to ertapenem was determined using the broth microdilution method. According to the 2021 M100 guidelines issued by the Clinical and Laboratory Standards Institute (CLSI) (8), ertapenem resistance was defined as a minimum inhibitory concentration (MIC) ≥2 µg/mL, sensitive as MIC ≤0.5 µg/mL, and intermediate as MIC = 1 µg/mL. For meropenem and imipenem, resistance was defined as MIC ≥4 µg/mL, sensitive as MIC ≤1 µg/mL, and intermediate as MIC = 2 µg/mL. All CRKP strains were identified using matrix-assisted laser desorption/ionization time-of-flight mass spectrometry (MALDI-TOF MS) (Autof MS1000, Zhengzhou Antu Biological Engineering Co., Ltd.).

### Bacterial strain antimicrobial susceptibility testing

According to the “National Clinical Laboratory Operating Procedures” for routine culture, the isolated strains were identified using MALDI-TOF MS (Autof MS1000, Zhengzhou Antu Biological Engineering Co., Ltd.). Drug sensitivity testing was conducted using the bacteria identification and drug sensitivity instrument from Zhuhai Meihua Company. The breakpoints for each drug were determined according to the CLSI 2023 guidelines (8). The quality control strains used were *Escherichia coli* ATCC25922, *Klebsiella pneumoniae* ATCC700603, and *Klebsiella pneumoniae* ATCC13883 obtained from the National Center for Clinical Laboratories, Beijing, China.

### Phenotypic assays

The modified carbapenem inactivation method (mCIM) (9) was performed according to the CLSI guidelines. Phenotypic detection of ESBLs was carried out using the double disk synergy test according to the CLSI guidelines. The AmpC activity of the isolates was tested using a three-dimensional extraction method (10).

### Detection of multi-drug resistance genes

Polymerase chain reaction (PCR) was used to detect the most prevalent carbapenemase genes, including *bla*KPC, *bla*IMP, *bla*VIM, *bla*OXA-48, and *bla*NDM (11), as described elsewhere. ESBL genes (*bla_CTX-M_*, *bla_TEM_*, and *bla_SHV_*) (12–15) and plasmid-mediated AmpC genes (*bla_CIT_*, *bla_MOX_*, *bla_FOX_*, *bla_MIR_*, *bla_DHA_*, and *bla_ACC_*) (16) were analyzed. Moreover, genes related to the efflux pump (*acrAB*, *acrA*, *kdeA*, *ketM*, *kpnE*, and *tolC*) (17, 18) and OMP genes (*ompK35* and *ompK36*) (17) were examined. Positive PCR amplification products were sequenced, and the DNA sequences were compared with reported nucleotide sequences in the National Center for Biotechnology Information (NCBI) database.

### Quantitative real-time PCR for efflux pump and porin gene expression

Quantitative real-time PCR (qRT-PCR) was performed to evaluate the transcriptional expression levels of OMP genes and efflux pump genes. Primer sequences were adopted from previously published studies (19). The *rpoB* gene was used as the internal reference. Total RNA was extracted using the chloroform–isopropanol method, followed by qRT-PCR analysis. All reactions were conducted in triplicate, and fold changes in gene expression were calculated as previously described (5). The *Klebsiella pneumoniae* ATCC 13883 strain was used as the positive control for assessing target gene transcription, and the results were expressed using the 2^−ΔΔCt method.

### Outer membrane protein isolation and sodium dodecyl-sulfate polyacrylamide gel electrophoresis (SDS-PAGE)

Outer membrane purification was performed following modifications of the methods used by Carlone et al. (20) and Hernández-Allés et al. (21). After inoculating the strain to be tested onto Mueller Hinton (MH) agar plates, single colonies were picked and inoculated into Luria-Bertani broth (LB broth) and then grown to the logarithmic phase. Bacterial precipitates were washed twice with 10 mM phosphate-buffered saline (PBS) and then subjected to five freeze-thaw cycles at –80°C. The bacteria were ultrasonically disrupted on ice, and the supernatant was centrifuged at 5,000 rpm for 10 min at 4°C. The resulting supernatant was centrifuged at 18,000 rpm for 60 min at 4°C, and the pellet was suspended in 2% sodium dodecyl sarcosinate PBS for 30 min at room temperature. This mixture was centrifuged at 18,000 rpm for 30 min at room temperature and washed twice with 1% sodium dodecyl sarcosinate. The precipitate was washed twice with 1% sodium dodecyl sarcosinate. The final precipitate containing the bacterial outer membrane protein was resuspended by adding 100 µL of 10 mM PBS (pH 7.0) and then stored at –80℃ for further use.

The outer membrane proteins were thoroughly mixed with the sampling buffer and boiled for 5 min before being subjected to SDS-PAGE. After 120 min of electrophoresis, the proteins were stained with Coomassie Brilliant Blue. Differences in the expression of outer membrane proteins between the clinical isolates and the standard strains were then compared.

## RESULTS

### Bacterial isolates, antimicrobial susceptibility, and molecular epidemiology

**Fig 1:**
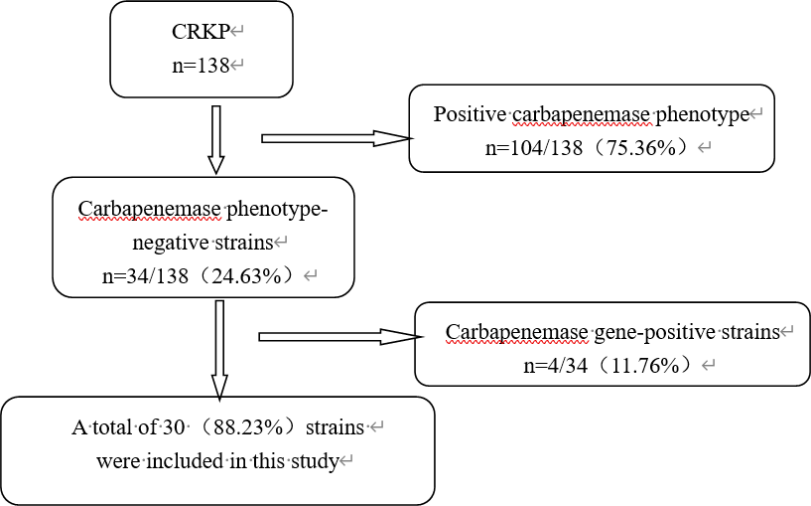
Isolation and processing of clinical strains.

Carbapenemase phenotyping was performed in 138 CRKP strains from various clinical sources. Of these, 108/138 (78.26%) were carbapenemase-positive. After validating the carbapenemase gene, 30 (21.74%) strains were included in the study. The isolation and processing of clinical strains are illustrated in Fig. 1. Most KP strains were isolated from sputum and alveolar lavage samples (36.66%, *n* = 11), followed by urine specimens (23.33%, *n* = 7) and blood (13.33%, *n* = 4). The patients were predominantly male, ranging in age from 4 to 91 years. The strains were mainly isolated from organ transplantation (20%, *n* = 6) and intensive care medicine (20%, *n* = 6) departments, followed by hematology (10%, *n* = 3). The collected strains exhibited a high degree of resistance to various antimicrobial drugs. ETP resistance and intermediate rates were high at 100%, while resistance rates to IPM and MEM were low at 3.33% and 10.00%, respectively. Resistance to all β-lactams exceeded 70%, and resistance to fluoroquinolones and cotrimoxazole was higher than 75%. Sensitivity to both tigecycline and polymyxin B was high. The antimicrobial susceptibility results of the strains are shown in Table 1.

**TABLE 1:** Resistance rate of non-carbapenemase-producing CRKP strains to antibiotics

Antibiotic	R (%)	I (%)	S (%)
Ertapenem	19(63.33)	11(36.67)	0(0.00)
Imipenem	1 (3.33）	1 (3.33）	28 (93.33）
Meropenem	3 (10.00）	2 (6.67）	25 (83.33）
Cefazolin	30(100.00)	0 (0.00）	0 (0.00）
Cefuroxime	30(100.00)	0 (0.00）	0 (0.00）
Cefotaxime	28 (93.33）	1 (3.33）	1 (3.33）
Ceftazidime	29 (96.67）	0 (0.00）	1 (3.33）
Cefepime	22 (73.33）	2 (6.67）	6 (20.00）
Aztreonam	24 (80.00）	0 (0.00）	6 (20.00）
Cefoxitin	23 (76.67）	3 (10.00）	4 (13.33）
Ampicillin/sulbactam	27 (90.00）	2 (6.67）	1 (3.33）
Amoxicillin/clavulanic acid	29 (96.67）	0 (0.00）	1 (3.33）
Piperacillin/tazobactam	15 (50.00）	11 (36.67）	4 (13.33）
Cefoperazone/sulbactam	20 (66.67）	2 (6.67）	8 (26.66）
Ticarcillin/clavulanic acid	28 (93.33）	1 (3.33）	1 (3.33）
Gentamicin	18 (60.00）	0 (0.00）	12 (40.00）
Amikacin	10 (33.33）	2 (6.67)	18 (60.00)
Tobramycin	17 (56.67)	5 (16.67)	8 (26.66)
Minocycline	16 (53.33)	6 (20.00)	8 (26.66)
Levofloxacin	24 (80.00)	6 (20.00)	0 (0.00)
Ciprofloxacin	25 (83.33)	5 (16.67)	0 (0.00)
Moxifloxacin	30 (100.00)	0 (0.00)	0 (0.00)
Cotrimoxazole	25 (83.33)	0 (0.00)	5 (16.67)
Nitrofurantoin	1 (3.33)	3 (10.00)	3 (10.00)
Chloramphenicol	24 (80.00)	1 (3.33)	5 (16.67)
Tigecycline	0 (0.00)	4 (13.33)	26 (86.67)
Polymyxin B	1 (3.33）	29 (96.67）	0 (0.00）

### Phenotyping test results

Among the 30 non-carbapenemase-producing CRKP strains, 73.33% (22/30) were ESBL phenotype-positive, while 26.67% (8/30) were ESBL phenotype-negative strains. In addition, 26.67% (8/30) were AmpC phenotype-positive, while 73.33% (22/30) were AmpC phenotype-negative strains.

### Characterization of antimicrobial resistance genes

ESBL resistance genes were detected in all 30 non-carbapenemase-producing CRKP strains. TEM genes were detected in 27 strains (90%), SHV genes in 28 strains (93.33%), CTX-M-2 genes in four strains (13.33%), CTX-M-9 in five strains (16.67%), and CTX-M-15 genes in 18 strains (60%). CTX-M-8 and CTX-M-25 genes were not detected. In addition, 13 strains (43.33%) carried AmpC resistance gene, all of which were DHA2. The detailed results are shown in Table 2.

**TABLE 2:** A β-lactamase genotype was observed in *Klebsiella pneumoniae* collected

β-lactamase genotype	Number of isolated strains
SHV	1 (3.33%)
TEM, SHV	1 (3.33%)
TEM, CTX-M-15	1 (3.33%)
TEM, SHV, CTX-M-15	12 (40%)
TEM, SHV, CTX-M-2, CTX-M-9	2 (6.67%)
SHV, DHA2	1 (3.33%)
SHV, CTX-M-15, DHA2	1 (3.33%)
TEM, CTX-M-15, DHA2	1 (3.33%)
TEM, SHV, CTX-M-15, DHA2	2 (6.67%)
TEM, SHV, CTX-M-2, CTX-M-15, DHA2	1 (3.33%)
TEM, SHV, CTX-M-2, CTX-M-9, DHA2	2 (6.67%)
TEM, SHV, DHA2	5 (16.67%)
Grand Total	30

### Expression of OMP genes and efflux pump genes

**Fig 2:**
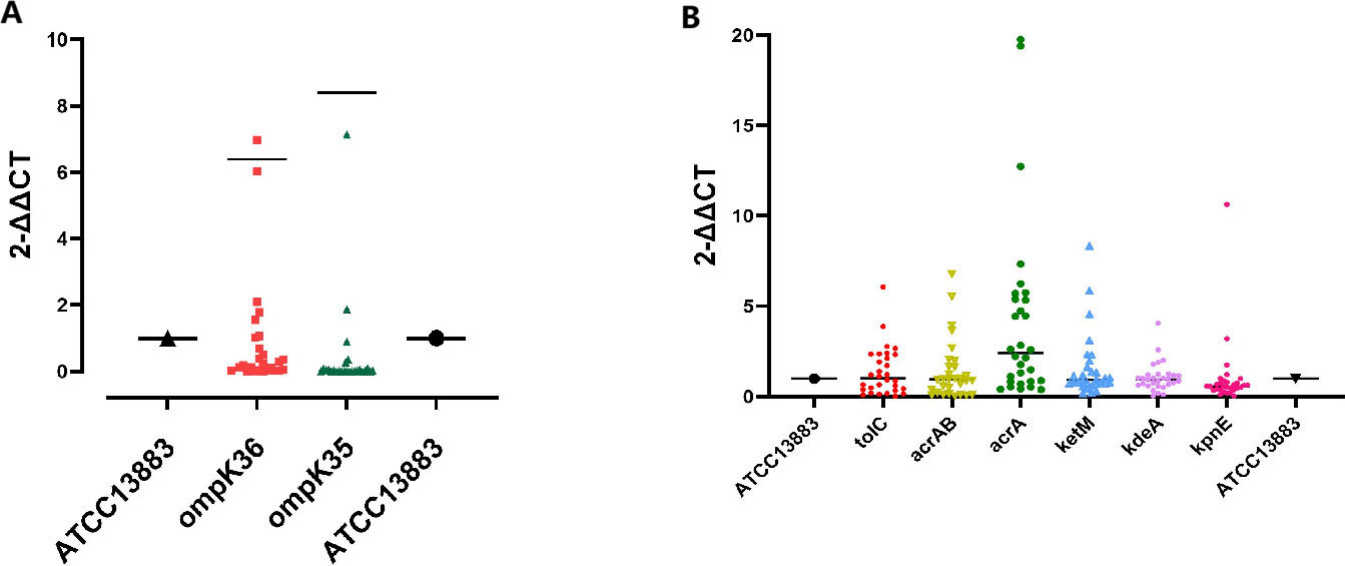
Transcription levels of porin genes OmpK35 and OmpK36 and efflux pump genes. The transcription levels (2^−△△Ct^ values) of the porin genes OmpK35 and OmpK36, as well as the efflux pump genes tolC, acrAB, acrA, ketM, kdeA, and kpnE, in the standard strain ATCC 13883 were set to 1. The 2^−△△Ct^ values of the test samples were compared with 1: values greater than 1 indicate upregulation of gene transcription levels, while values less than one indicate downregulation. (A) porin transcript level results; (B) transcript level results of efflux pump genes

The relative expression levels of outer membrane proteins (ompK35 and ompK36) and efflux pump genes (tolC, acrAB, acrA, ketM, kdeA, and kpnE) were measured by real-time PCR and calculated using the 2^^−ΔΔCT^ method. The experiment was repeated three times. The mean relative expression levels and standard deviations (SD) are presented. Compared with the reference strain ATCC 13883, the expression levels of ompK35 and ompK36 were significantly decreased in most of the clinical isolates (20/30), suggesting reduced OMP-mediated permeability. The fold changes of tolC, acrAB, acrA, ketM, kdeA, and kpnE were 1.39 ± 1.34, 1.44 ± 1.64, 4.17 ± 5.01, 1.56 ± 1.79, 1.07 ± 0.79, and 1.02 ± 1.90, respectively, suggesting that efflux pump activity may be enhanced (Fig. 2)

### OMP analysis

**Fig 3:**
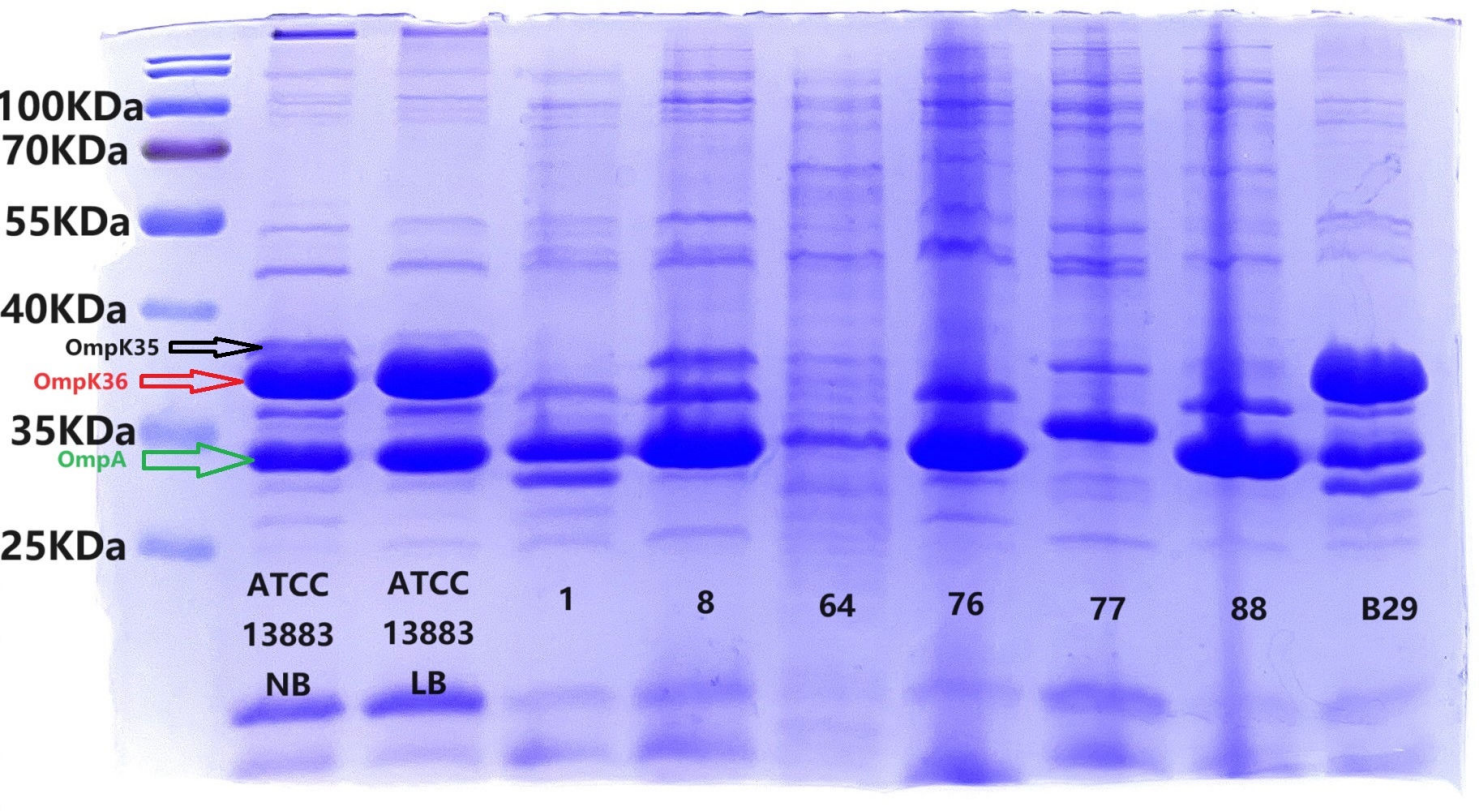
SDS-PAGE analysis of OMPs of some strains. Each bacterial isolate was cultivated in both high-osmolarity medium, Luria Broth (LB), and low-osmolarity medium, Nutrient Broth (NB). Low-osmolarity conditions are favorable for the optimal expression of the OmpK35 protein. In LB, the OmpK36 protein (red arrow) is expressed at a higher level than in NB, where both OmpK35 (black arrow) and OmpK36 are expressed in ATCC13883. OmpA, the most abundant outer membrane protein in the Enterobacteriaceae, is present in all isolates (green arrow). Strain 1 lacks both ompK35 and ompK36; strain 8 lacks ompK36; strain 76 lacks ompK35, and the expression of ompK36 protein is decreased.

The ompK35 and ompK36 genes of 30 *Klebsiella pneumoniae* isolates were PCR-amplified and sequenced. Sequence comparison with reference strains *K. pneumoniae* AJ011501(GenBank accession no. AJ011501.1) and FJ577673 (GenBank accession no. FJ577673.1) revealed varying degrees of nucleotide deletions and point mutations. Subsequent SDS-PAGE analysis demonstrated that the majority of ESBL-producing isolates lacked OmpK35 expression (26/30), and 66.67% (20/30) exhibited either absence or aberrant expression of OmpK36. Notably, 26.67% (8/30) of the isolates lacked both porins. In three isolates, aberrant protein bands were observed at unexpected molecular weights: isolates 1 and B29 exhibited bands at ~33 kDa, while isolate B17 showed a distinct band at ~25 kDa. Representative SDS-PAGE profiles of selected non-carbapenemase-producing CRKP isolates, in comparison with the reference strain *K. pneumoniae* ATCC 13883, are shown in Fig. 3.

Furthermore, we assessed the effect of osmolarity on OmpK35 expression in *K. pneumoniae* ATCC 13883. Under both low- and high-osmolarity conditions, OmpK35 was expressed; however, SDS-PAGE revealed a more distinct band under low-osmolarity conditions, suggesting enhanced expression or stability of OmpK35 under these conditions.

## DISCUSSION

In this study, we explored the resistance mechanism of non-carbapenemase-producing CRKP to aid in the clinical diagnosis and treatment of CRKP-induced infections. The 30 strains in this study were mainly isolated from the departments of organ transplantation and critical care medicine. Similarly, Hamzaoui et al. study found that most strains originated from intensive care medicine departments, with a predominance of urine-sourced specimens (7). Likewise, Wise et al. (22) found that non-carbapenemase-producing CRKP was predominantly of urinary tract origin. Organ transplantation departments often involve surgical procedures, pre-existing and long-term immunosuppressive therapy, invasive procedures, and the use of related carbapenem antibiotics, all of which increase the risk of infection (23). Patients in critical care units typically have compromised immune systems, underlying diseases, prolonged hospital stays, extensive histories of broad-spectrum antibiotic use, and are subjected to multiple invasive procedures, further increasing their infection risk (24). Therefore, strengthening monitoring in key departments and reducing CRKP-related infections is crucial.

In the present study, all non-carbapenemase-producing CRKP strains were found to be resistant or intermediate to ertapenem. In addition, three strains were resistant to meropenem, one strain was resistant to imipenem, and all exhibited a multi-drug resistance phenotype. This included high levels of resistance to most antimicrobial drugs, such as cephalosporins, aztreonam, quinolones, and β-lactams/β-lactamase inhibitor combinations. Resistance gene testing revealed that all strains carried either ultra broad-spectrum β-lactamase or AmpC-type β-lactamase genes, with 46.67% (14/30) carrying both ESBLs and AmpC enzyme resistance genes. High expression of AmpC enzyme or the presence of ESBLs alone typically does not lead to carbapenem resistance. However, when membrane permeability is reduced, these strains can simultaneously hydrolyze carbapenem antibiotics and prevent their entry into the bacterium, resulting in resistance to carbapenem antibiotics (25). All the strains were highly resistant to ertapenem.

A total of 36.33% (10/30) of the isolates showed high-level resistance to ertapenem and lacked both ompK35 and ompK36, suggesting that the susceptibility to ertapenem was greatly affected by changes in porin (Table 3). Previous studies have shown that most ESBLs and AmpC enzyme-producing KP are sensitive to ertapenem but become resistant in the absence of membrane pore protein strains (26–28). This suggests that resistance to ertapenem is more variable compared with imipenem and meropenem (22, 29). Previously reported outbreaks of ertapenem-resistant, CTX-M-15-producing isolates of KP were mainly due to early termination of CTX-M-15-producing isolates of KP at amino acid position 63, preventing the proper expression of ompK35, and a mutation in the L3 loop of ompK36(30). In the Yuan et al. study on strain 2018B120, which showed resistance to ertapenem but susceptibility to meropenem and imipenem, the genomic analysis revealed that the gene coding for antimicrobial resistance resulted in a high level of resistance to β-lactam antibiotics, aminoglycosides, and quinolones and that the loss-of-function mutations in ompK35 and ompK36 were the main causes of ertapenem resistance (31). The strains expressing ESBLs in this study may respond to antibiotic pressure by losing membrane porins or by simultaneously increasing ESBL expression, thereby reducing antibiotic uptake or increasing antibiotic degradation. The high resistance to most antimicrobial agents observed in this study may be due to the combined effect of ESBL enzyme expression and loss of membrane porins. Most studies have confirmed the effect of ompK36 on the antibiotic permeability of carbapenemases (27, 32, 33). However, the contribution of ompK35 is still uncertain, with some researchers suggesting it does not play a significant role. Studies have shown that most ESBL-producing KP lack ompK35. The absence of ompK35 is considered crucial in contributing to drug resistance and may indirectly support other resistance mechanisms, such as the absence of ompK36 or enhanced bacterial exocytosis (21, 34). Strains with deletion of both membrane pore proteins showed significantly higher MICs to carbapenems, suggesting that ompK35 and ompK36 play important roles in carbapenem resistance development in KP (7). Recent research has also identified other outer membrane proteins, such as ompK26(35), which are connected to carbapenem resistance in KP. The abnormal protein of about 33 kDa expressed in strain one and strain B29 requires further investigation to determine its relation to carbapenem resistance. These strains may also harbor other resistance mechanisms, such as uncommon carbapenemase genes, a reduced number of PBSs, or mutations. In addition, the combined overexpression of efflux pumps and the presence of abnormal proteins in these strains should be explored as a potential resistance mechanism.

**TABLE 3:** Relationship between ompK35 and ompK36 membrane porin expression and ETP MICs

MIC (mg/L)	Both loss	Loss of ompK35	Variation in ompK35 expression	Loss of ompK36	Variation in ompK36 expression	Normal expression
ETP >4 (*n* = 10)	6	0^*a*^	0^*a*^	1	3	0^*a*^
ETP = 4 (*n* = 5)	0^*a*^	2	0^*a*^	1	2	0^*a*^
ETP = 2 (*n* = 4)	1	2	0^*a*^	0^*a*^	1	0^*a*^
ETP = 1 (*n* = 11)	1	4	0^*a*^	0^*a*^	4	2
Total (*n* = 30)	8	8	0^*a*^	2	10	2

^*a*^ 0 indicates that no isolates with this characteristic were detected.

In this study, we assessed the transcriptional levels of porin genes by qPCR. Compared with the standard KP strain ATCC 13883 (expression level = 1), most CRKP strains showed reduced mRNA levels of ompK35 and ompK36, indicating decreased gene expression. These findings suggest that porin loss or downregulation may contribute to carbapenem resistance by limiting drug influx. However, strains B15, B3, and B4 exhibited relatively high transcriptional levels of porin genes, indicating that porin-mediated permeability might be partially preserved in these isolates. Notably, these strains also showed upregulated expression of efflux pump genes, including tolC, acrAB, ketM, kdeA, and kpnE, suggesting that even with relatively intact porin function, bacteria may rely on efflux systems as a compensatory resistance mechanism to reduce intracellular antibiotic concentrations. This highlights the diversity of resistance strategies in CRKP strains and the potential synergism between porins and efflux pumps in mediating drug resistance.

The detection rate of efflux pump genes in this study was high, with 100% of strains carrying kdeA, ketM, and kpnE genes. The carriage rates for acrAB, acrA, and tolC were over 65%, and 60% (18/30) of the strains had efflux pump genes detected. The strains were primarily sourced from organ transplantation and intensive care units, which frequently use carbapenem antibiotics, thus enhancing the bacterial efflux pump system activity (36). The currently known efflux pumps associated with carbapenem resistance in KP are acrAB-tolC, kdeA, kpnE, and kpnGH (37). Active efflux pump-mediated resistance typically does not cause high resistance levels, but when combined with other resistance mechanisms, it reduces drug effects and enhances drug efflux, restoring the efficacy of antibiotics (38). Filgona found that an efflux pump inhibitor reduced the MIC value of CRKP to carbapenems like ertapenem, thus involving efflux pumps in carbapenem resistance (39). Seecoomar noted that the ArcAB efflux pump in carbapenemase-producing Enterobacteriaceae bacteria was associated with β-lactam resistance (40). In addition to gene detection, this study also evaluated the transcriptional expression levels of several key efflux pump genes. Among them, *acrA* showed the most pronounced upregulation, suggesting that efflux pump activity may be enhanced in some strains. Although expression levels varied between isolates, these results support the notion that efflux pumps may play a contributing role in reducing carbapenem susceptibility. Yazgan et al. provided evidence that the synergistic effect of carbapenemase production and a secondary resistance mechanism, such as upregulated efflux pump expression, can lead to carbapenem resistance (41). However, further functional validation is necessary to confirm their impact on phenotypic resistance. The present study only preliminarily detected efflux pump gene carriage and transcriptional changes; efflux pump activity at the protein level and its correlation with clinical resistance phenotypes will be a focus for future research.

Taken together, our findings suggest that ertapenem resistance in non-carbapenemase-producing *K. pneumoniae* results from the combined action of ESBL or AmpC production, porin deficiency, and efflux pump overexpression. All isolates carried β-lactamase genes, indicating a foundational role in resistance. Porin loss or mutation was found in 86.7% of isolates, and 26.7% lacked both OmpK35 and OmpK36, highlighting the key role of reduced permeability. Efflux pump gene upregulation, particularly acrA, was also observed in over half of the strains, suggesting a compensatory mechanism when porin function is partially retained. These overlapping mechanisms work synergistically to confer high-level resistance, underscoring the need for integrated molecular diagnostics to guide effective treatment strategies.

By analyzing the resistance mechanisms of non-carbapenemase-producing CRKP isolated from various clinical departments, we examined the presence of resistance genes, porin genes, and efflux pump genes. Additionally, we assessed the transcription levels and expression of porin and efflux pump genes, confirming that these CRKP strains primarily produce ESBLs and AmpC. In addition, the lack of expression of ompK35 and the loss or decreased expression of ompK36 were combined with various efflux pump genes. Our study provides a scientific foundation for understanding the spread and treatment of CRKP, contributing to hospital infection prevention and control efforts. However, certain limitations should be acknowledged. First, the relatively small sample size may not fully represent the bacterial diversity in the Nanning region. Second, the absence of Western blot analysis for outer membrane porins prevents direct confirmation of protein expression levels, which would have strengthened the correlation between porin loss and carbapenem resistance. Third, the lack of whole-genome sequencing limits our ability to comprehensively analyze resistance mechanisms and genetic variations. Future studies should incorporate larger sample sizes, Western blot validation, and whole-genome sequencing, along with *in vivo* and *in vitro* experiments, to further elucidate the resistance mechanisms of non-carbapenemase-producing CRKP.

### Conclusion

In summary, this study demonstrates that ertapenem resistance in non-carbapenemase-producing *K. pneumoniae* is primarily mediated by a combination of ESBL or AmpC production, porin loss or decreased expression, and efflux pump upregulation. Among these, porin deficiency—particularly dual loss of OmpK35 and OmpK36—appears to play a pivotal role, while efflux activity may act as a compensatory mechanism.

These findings highlight the multifactorial and synergistic nature of carbapenem resistance in the absence of carbapenemases. Given the growing prevalence of such strains, especially in high-risk clinical settings, there is an urgent need for routine surveillance that includes molecular characterization of resistance determinants. A better understanding of these mechanisms can guide more rational antibiotic use and inform the development of targeted treatment strategies to curb the spread of CRKP.

## Data Availability

Data will be made available on request.

## SUPPLEMENTAL MATERIAL

Supplemental material jb.00148-25-s0001.xls Results for all strains.

ASM does not own the copyrights to Supplemental Material that may be linked to, or accessed through, an article. The authors have granted ASM a non-exclusive, world-wide license to publish the Supplemental Material files. Please contact the corresponding author directly for reuse.
